# Barriers and facilitators in access to cervical cancer secondary prevention in Cochabamba, Bolivia: a qualitative study of healthcare providers’ perceptions

**DOI:** 10.1186/s12875-026-03422-2

**Published:** 2026-06-15

**Authors:** Carla Huanca Challgua, Daniel Eid Rodriguez, Isabel Goicolea, Ida Linander

**Affiliations:** 1 Department of Epidemiology and Global Health, Umeå University, Umeå, Sweden; 2 Biomedical and Social Research Institute, Faculty of Medicine, Universidad Mayor de San Simón, Cochabamba, Plurinational State of Bolivia

**Keywords:** Cervical cancer, Screening, Barriers and facilitators, Bolivia, Healthcare providers

## Abstract

**Background:**

Despite the availability of cervical cancer (CC) screening in Bolivia, coverage remains low and uneven across municipalities. This suggests the presence of barriers to effective secondary prevention, which involves early detection through screening and treatment of precancerous lesions. There is limited research on the perceptions of healthcare providers (HCPs) regarding the challenges that women in Bolivia face when accessing CC secondary prevention. This study therefore explored HCPs’ perceptions of barriers and facilitators for women’s access to CC secondary prevention in Cochabamba, Bolivia.

**Methods:**

Qualitative interviews were conducted with 30 HCPs working in CC secondary prevention, including gynaecologists, general practitioners, nurses, and auxiliary nurses. The interviews were analysed using reflexive thematic analysis. Levesque’s access to healthcare framework informed the study design, and the socio-ecological model was used to discuss the findings across multiple levels.

**Results:**

HCPs perceived numerous barriers and limited facilitators in access to CC secondary prevention at different levels. Barriers at the individual level included information gaps among women. Incentive-based campaigns were used to increase screening, but these did not address the actual treatment barriers or root causes. At the interpersonal–community level, barriers included family-transferred misconceptions, gender norms, and women’s fear of intimate partner violence if attending screening. Facilitators included the use of the Quechua language during healthcare encounters, and material incentives to encourage attendance. Barriers at the organizational–structural level included a lack of clarity regarding which women should be screened and how often, the concentration of centralized cytology services in a single laboratory, unclear responsibilities among staff for sample collection and result delivery, and bureaucratic and administrative barriers that limited access to screening and timely results. These barriers led to long waiting times, generating mistrust and reluctance to engage with CC screening.

**Conclusions:**

HCPs perceive barriers to CC secondary prevention at the individual, community, and organizational levels, including women’s lack of information, family-driven misconceptions, gender norms, unclear screening guidelines, centralized cytology services, and bureaucratic delays that erode trust. Facilitators include the use of Quechua language during care and material incentives to encourage screening attendance; however, these do not address treatment barriers or structural causes.

**Supplementary Information:**

The online version contains supplementary material available at 10.1186/s12875-026-03422-2.

## Introduction

Cervical cancer (CC) is the fourth most common cancer in women, with around 660,000 new cases in 2022. In the same year, about 94% of the 350,000 deaths caused by CC occurred in low- and middle-income countries [1]. Although the burden of CC has decreased in some countries, there are still significant differences between and within countries [1]. Latin America and the Caribbean constitute the region with the second highest incidence (15.1 per 100,000 women-years) and mortality rates (7.7 per 100,000 women-years) after Africa (26.4 and 17.6 per 100,000 women-years, respectively) [2]. Within South America, Bolivia accounted for the highest CC incidence rates (38.7 per 100,000 women-years) and mortality rates (18.3 per 100,000 women-years) in 2022 [2]. Regional differences in the CC burden are related to inequities in access to vaccination, screening, and treatment services, risk factors including HIV prevalence, and social and economic determinants [3].

CC is one of the most preventable and curable forms of cancer when detected and treated early. Precancerous lesions can progress to CC in 15–20 years in immunocompetent women or 5–10 years in women with weakened immune systems. CC offers ample opportunity for prevention and treatment if detected early. CC secondary prevention involves early detection through screening and treatment of precancerous lesions [4]. Cytology-based screening through Pap smears has reduced CC mortality in many European countries. However, methodological and operational limitations have hampered the impact in low- and middle-income countries [5]. The World Health Organization recommends using human papillomavirus (HPV) testing as the primary screening test [6]. While some high and middle-income countries have transitioned to HPV testing, cytology-based screening is still the main strategy in South America [7].

In Bolivia, the current national CC screening guideline from 2024 recommends Pap smear screening in women aged 25–70 with a three-yearly screening interval or visual inspection with acetic acid (VIA) annually in those aged 30–55 [8]. Although HPV testing has been carried out recently in some health centres, it has not yet been implemented nationwide [9, 10]. CC screening services are offered mainly in public primary care and secondary facilities for free, but women can also pay for screening at non-governmental and private facilities. Despite the availability of free public services, the overall screening coverage through Pap and VIA remains low, with only an average of 14% of women aged 25–69 being screened in 2022, including tests performed within the public, non-governmental, and private services. Large inequities in CC screening coverage have been shown between departments and municipalities. Some municipalities have reported 59% coverage, while others report rates below 1%; this might reflect both geographic barriers and data limitations [11]. Nevertheless, low CC screening rates could suggest the existence of major barriers in the screening program.

Previous studies conducted in Ecuador, Peru, and Colombia, which are all South American countries in the neighbourhood of Bolivia, have identified the existence of barriers in the implementation of CC screening programs. From the perspective of healthcare providers (HCPs), these include fragmented health systems, weak information flow between levels of care, insufficient investment in screening programs, and shortages of trained personnel and resources [12, 13]. Barriers reported from the perspective of women include difficulties in scheduling appointments and obtaining follow-up care, distance to health services, financial and logistical constraints, and poor communication with HCPs [14, 15].

In Bolivia, the few existing studies on CC secondary prevention have shown barriers such as cultural misconceptions, low risk perception, taboos around Pap testing, distrust of nurses, and discomfort with male HCPs. Poor service delivery, shortages of trained staff, inadequate infrastructure and training, long waiting times, high costs, and delays in receiving results have also been identified as barriers by women, community members, and HCPs [16–18]. However, there remains a gap in understanding the persistence of these barriers and how providers manage and respond to health system constraints in their daily work. These findings underscore the need to complement and update previous studies by exploring the perspectives of HCPs who work face-to-face with women and navigate the health system. This study therefore aimed to explore HCPs’ perceptions of barriers and facilitators for women’s access to CC secondary prevention in Cochabamba, Bolivia.

### Theoretical framework

The analysis was guided by two complementary frameworks: Levesque’s patient-centred access to healthcare framework [19] and Daley et al.’s socio-ecological model [20]. Levesque’s framework conceptualizes access as interconnected stages shaped by both the organization of healthcare and the population’s ability to seek and use care. It includes five key dimensions: approachability (healthcare services are known to users and can be obtained by them), acceptability (healthcare services meet the social and cultural norms and needs of users), availability (healthcare is organized to meet users’ needs in terms of time, location, and services), affordability (service fees correspond to the ability and willingness of the users to pay), and accommodation (appropriateness and the structure and process of providing care, including the quality of the facility and the consultation) [19].

Daley et al. applied a socio-ecological model to study barriers to CC screening and treatment, showing how the interconnected barriers operate across and within the various levels: the political level (national policies and funding), the organizational level (health system resources and structures), the community level (cultural values, social norms, inter-organizational relationships), the interpersonal level (family and social networks), and the individual level (knowledge and attitudes) [20].

One previous example of applying these frameworks to research on CC prevention is a systematic review that examined the implementation of decentralized CC prevention services across African settings [21]. Another study, from the Swedish context, combined both frameworks to understand the access of young migrants to sexual and reproductive health services, but not specifically CC prevention [22]. These frameworks were chosen to provide a chronological and layered perspective to examine how HCPs perceive women’s access to CC secondary prevention services.

### Methods

This qualitative study was based on interviews conducted with 30 HCPs working with CC secondary prevention in Cochabamba, Bolivia.

### Study setting

Cochabamba is the third most populated department in Bolivia, with an estimated 2 016 357 inhabitants as of 2024, of whom approximately 1 421 617 (70.5%) are concentrated in urban areas and 594 740 (29.5%) in rural areas [23]. It is administratively divided into 47 municipalities and one autonomous Indigenous government (GAIOC in Spanish), Raqaypampa. The department is also divided into 5 regions: Metropolitan, Southern Cone, Andean, Tropical, and Valleys [23]. Bolivia has 36 Indigenous languages; one of the most predominant is Quechua, with 1.39 million Quechua speakers throughout the country, of whom 569 072 live in Cochabamba. Among this population, 40.6% in urban areas and 67.2% in rural areas are bilingual and speak an indigenous language [23].

In 2024, the Bolivian Ministry of Health developed the National Clinical Care for Cervical Cancer guideline and established free-of-charge CC secondary prevention within the public sector. This preventive effort formed part of the universal healthcare coverage (SUS in Spanish) [8] which was implemented in 2019 to guarantee universal and free access to healthcare in the public sector for individuals lacking other forms of health insurance. Around 52.19% of the Bolivian population is registered in the SUS [24].

The Departmental Health Service of Cochabamba is administratively organized into 14 healthcare networks, comprising a set of primary, secondary, or tertiary health facilities generally grouped within municipalities [25]. As of 2024, the health system in Cochabamba consisted of 563 primary care facilities, 58 secondary care hospitals, and 20 tertiary care hospitals. These facilities are distributed across the public sector, the social security system, and private providers. Primary care is predominantly delivered through the public sector, which administers 472 of the 563 primary care services, making it the largest provider in the department [25].

Once Pap or VIA samples are taken, they are sent for analysis to laboratories generally located in secondary or tertiary-level facilities. The guidelines do not specify which healthcare professionals should perform the screening. When test results indicate abnormalities or precancerous lesions, women are referred from primary to secondary care. Another key component of CC secondary prevention is treatment access for women with precancerous lesions. According to the guideline, colposcopies should be performed at secondary-level hospitals, but in practice, few hospitals have colposcopes. Tertiary-level hospitals provide more specialized CC diagnostics and treatment [8].

In 2008, the Bolivian Ministry of Health introduced the Intercultural Community Family Health Model (SAFCI in Spanish), a health policy that acknowledges the cultural and organizational diversity of Indigenous and rural populations, and promotes interculturality, community participation, and strengthening of primary care [26]. One aim of SAFCI is to integrate biomedical and Indigenous health knowledge and strengthen the connection between health services and communities. It uses a family-centred approach and involves home visits, facilitating health promotion and disease prevention [26]. However, the guidelines lack clarity regarding the implementation of SAFCI in relation to CC secondary prevention.

### Recruitment

Thirty participants were recruited using purposive sampling with the aim of capturing a diverse range of perspectives from HCPs involved in CC secondary prevention within public sector facilities of Cochabamba. To ensure variation across geographic areas and administrative contexts, facilities were selected from each of the 14 healthcare networks in the department. Initial contact with the facility directors was made by the first author (CH). Formal letters of invitation to participate and consent forms describing the study were sent to the directors, who assisted in identifying eligible HCPs for participation based on their involvement in CC screening and related services within primary, secondary, and tertiary care. Laboratory staff were excluded.

### Data collection

Data were collected using a semi-structured interview guide that was collaboratively developed and refined by all the authors. CH conducted most of the interviews, while the remaining 7 were conducted by a research assistant. The interview guide covered the following key areas: health system response to CC secondary prevention, barriers and facilitators, and the encounter and communication between health personnel and women seeking care (Supplementary File 1).

A first round of 7 interviews was conducted in August 2023. Based on preliminary analysis of these interviews, the interview guide was slightly revised to improve the interview flow and deepen emergent themes. The remaining interviews were conducted between October and November 2024 in the HCPs’ workplaces. All interviews were in Spanish.

The HCPs received information about the project before agreeing to participate, and signed their written informed consent before being interviewed (Supplementary File 2). The interviews lasted between 30 min and 1 h. All interviews were audio-recorded and transcribed verbatim using the offline NoScribe software, and were then cross-checked for accuracy by the first author. No identifying information was included in the transcripts.

### Participants

The 30 participants comprised 20 women and 10 men working in different facilities at the primary, secondary, and tertiary levels. One to three participants were interviewed from each of the 14 healthcare networks. They were all involved in CC secondary prevention, working as gynaecologists, general practitioners, nurses, and auxiliary nurses (Table 1).

**Table 1:** Characteristics of the participating healthcare providers (HCPs) (*n* = 30)

HCP characteristics	*n*
Age in years	
≤ 35	9
36–54	17
≥ 55	4
Gender	
Female	20
Male	10
Profession	
Gynaecologist	8
General practitioner	16
Nurse	3
Auxiliary nurse	3
Geographic location working place facilities	
Urban	9
Rural	21
Level of care	
Primary	22
Secondary	6
Tertiary	2
Years of practice as HCP	
< 5	7
5–15	14
> 15	9
Years working at the facility	
< 5	15
5–10	8
> 10	7

### Data analysis

The data were analysed using a reflexive thematic analysis inspired by the six-phrase framework described by Braun and Clarke [27]. We started with in-depth repeated reading of the transcripts before coding the material. Codes were generated inductively, and the data were managed using NVivo software. The transcripts were coded by CH, and discussed regularly with IL, IG, and DE for refinement. This process included repeated meetings within the research team to discuss the analysis and preliminary themes. Through an iterative process of writing, going back and forth between the transcripts and codes, and team discussions, the subthemes and themes were identified.

Levesque’s access to healthcare framework [19] informed the initial design of the study, helping structure the exploration of women’s pathways through CC secondary prevention from the HCPs’ perspectives. The socio-ecological model [20] was later used to deepen the understanding of the findings across multiple levels: individual, interpersonal–community, and organizational–structural.

## Results

The HCPs’ perceptions of the barriers and facilitators for CC secondary prevention are presented below in three themes and their associated subthemes (Table 2). Before delving into the themes, we want to highlight the effects of these barriers to CC secondary prevention. During the interviews, multiple HCPs shared stories of women who arrived at the facility too late, when CC had already progressed beyond the point of being treatable or curable. One provider described a young mother of four:

*A patient with a lot of pain and bleeding during sexual intercourse came. We took a Pap smear and I saw it was cancer. The patient was young*,* 38–39 years old*,* and had four children. Unfortunately*,* she passed away two weeks later.* (HCP25)

**Table 2:** Overview of themes and subthemes

Themes	Subthemes
Negotiating participation: campaigns and incentives to overcome sociocultural and gendered barriers?	Women’s beliefs, misconceptions, and responsibility Gender relations in healthcare and at home Community and incentive-based strategies for increasing screening uptake
Distance, economics, and language shaping access	Too far, too costly From misunderstanding to trust: the role of the Quechua language
Caught in bureaucracy: the costs of long waits	Universal healthcare in name, bureaucratic and opportunistic in practice The consequences and explanations of long waits

Another recounted the case of an elderly woman who had never been screened:

*An old woman came in. She was bleeding from her genitals. She had never had a Pap smear in her life. I took the Pap and she had advanced cancer*,* it had already metastasized*,* unfortunately she died.* (HCP9)

Similarly, one HCP reflected on the recurrence of such cases:

*This year two women in the community have already died from cervical cancer. One even went to La Paz for radiotherapy… Sometimes patients come at the last minute*,* when the cancer is already advanced and there isn’t much we can do.* (HCP19)

These testimonies illustrate how the failure of CC secondary prevention takes many forms: missed opportunities for timely diagnosis and treatment, women who were not screened due to their age, women coming too late with symptoms like bleeding or pain, and CC that had progressed beyond precancerous lesions. The following sections present our findings of how the HCPs explained the barriers that led to these situations and the limited strategies used in attempting to address them.

### Negotiating participation: campaigns and incentives to overcome sociocultural and gendered barriers?

The HCPs perceived a complex interplay of factors shaping women’s willingness to participate in screening, including misconceptions about CC, family and community influences, and gendered norms that undermined women’s comfort and autonomy. They explained that these barriers often translated into hesitation or resistance toward CC screening among women. To increase participation, they implemented a strategy of community campaigns and incentives to increase uptake, which might not directly target the sociocultural and gendered barriers but were seen as convincing women to take part in screening The question mark in this theme reflects this tension, raising questions about whether such strategies meaningfully address these barriers or primarily serve to compensate the low screening uptake.

#### Women’s beliefs, misconceptions, and responsibility

*We have a lot of difficulty with taking Pap smears because women don’t want to do it. When we offer [the Pap]*,* they say “Not today*,* I’ll come back another day*,*” but they never return… it’s difficult to convince them.* (HCP11)

A major challenge for the HCPs in CC secondary prevention was women’s hesitation or reluctance to undergo Pap smears. Reasons for such hesitation or avoidance were also mentioned. Some participants explained that women expressed suspicion about the Pap test, fearing that the procedure could cause pain or physical harm: *“They [women] tell you*,* ‘You want to infect me with something*,* you’re going to cut a piece of my uterus.”* (HCP14).

Several providers considered that misconceptions transmitted within families and community members also explained this hesitancy. Older relatives were described as influential in discouraging younger women from attending CC screening:

*Her [the patient’s] mother and mother-in-law told her*,* “Don’t get the Pap done because you won’t be able to have children*,*” …older women usually have a lot of beliefs.* (HCP9)

Some providers felt that low participation in CC screening was also linked to what they perceived as women being “careless” and not grasping the seriousness of CC: *“Unfortunately*,* our people are very careless*,* and when there is heavy bleeding and things get complicated*,* only then do they come to the health centre.”* (HCP17).

The participating HCPs also perceived limited knowledge and awareness about CC and its prevention among some women. This lack of understanding was often attributed to low educational levels and restricted access to health information:

*Most people here in the tropics region*,* because of their educational and sociocultural level*,* don’t know what cervical cancer is. There’s a great lack of information and awareness for prevention; we still need to work a lot on that.* (HCP2)

Finally, some HCPs observed that some women used traditional medicine when experiencing CC symptoms. It was often advice from neighbours that encouraged women to try herbal remedies. These practices were perceived by the HCPs as common in rural communities and contributing to delays in diagnosis and treatment.

*There are older [women] neighbours who tell them [women]*,* “I was like you too*,* take this herb or this other one*,*” and the patients believe in it*,* but they lose time*,* and when the symptoms become more marked*,* such as heavier bleeding*,* they return to the health centre.* (HCP15)

Overall, the HCPs described a set of individual and community-level barriers that they considered to be rooted in fear, mistrust, and traditional beliefs that they perceived to influence women’s engagement with CC secondary prevention and care.

#### Gender relations in healthcare and at home

*There are also women who are taking care of their animals or children*,* and cannot come to the health facility.* (HCP14)

The HCPs described how women’s participation in CC screening and seeking treatment was influenced by gender dynamics. In some rural areas, women’s domestic duties, such as taking care of animals or children, were mentioned as reasons for not attending health services. These obligations could also lead women to postpone or decline treatment, as illustrated by one provider working in primary care:

*We had an older patient with cervical lesions*,* but she refused to be referred [to continue with the treatment] because she told us she lived alone with her grandson. She had no one to leave him with.* (HCP17)

The HCPs also described how the gender relations at women’s homes influenced access to screening in yet another way, involving “machismo” (a Spanish word similar to sexism). They said that women often required their husband’s approval before undergoing a Pap test, particularly if the procedure was to be performed by a male provider:

*There is a lot of “machismo”. Husbands forbid women from being examined by a male doctor… when we offer to take the Pap*,* the women say*,* “I’ll ask my husband first.” […] They depend on their husbands*,* and it seems they don’t decide for themselves.* (HCP11)

This control affected the HCPs’ clinical practices. Several of them reported that men’s jealousy and distrust led them to adapt how they conducted examinations; for example, by inviting husbands or relatives to be present to prevent accusations of infidelity, as one male provider described:

*I don’t take the Pap alone*,* I always ask the nurse [to take the Pap]*,* or I tell the women to ask their husband or a relative to come in to avoid problems […] husbands are jealous and don’t want their wives being seen by anyone.* (HCP9)

The HCPs also described how women’s decisions to have a Pap smear or use contraception were shaped by a fear of intimate partner violence:

*They [women] always say*,* “My husband will scold me*,* he will hit me.” […] Some husbands control them [women] a lot and say that if their wives get Pap smears or use contraceptives*,* it’s because they want to be with other men.* (HCP4)

The providers also noted the challenges of framing CC as a sexually transmitted infection, as this can also place the responsibility and blame on women (for the infection), and can cause tension within couples:

*Talking about it [cervical cancer] as a sexually transmitted disease caused by the human papillomavirus is very complicated; husbands think it’s because of [women’s] infidelity […] there is a risk that the couple will fight over this*,* and those affected will be the family*,* especially the children.* (HCP20)

Additionally, the HCPs described how feelings of embarrassment and shame were common among women who were invited to take the Pap test, particularly when the examiner was a man. They mentioned that women in rural settings were often perceived as more “closed off”, and had a preference for female staff: *“Women in rural areas are a little more closed off; they have a little more confidence with female doctors than with male doctors[…] they feel embarrassed or ashamed to expose themselves to men.”* (HCP11).

Male providers described similar experiences, explaining that women’s discomfort frequently led them to seek assistance from female colleagues. Some HCPs described training nurses or other female staff to take the samples. One general practitioner explained: *“Since I’m a man*,* they don’t want me to take the Pap due to shame or modesty. So the nurse got trained to do it and support me*,* and she takes the Pap.”* (HCP9).

Overall, the HCPs perceived that gender dynamics in terms of domestic responsibilities, partner control, and fear of violence combined to create barriers related to family, husbands, and the clinical encounter. Strategies to counter this included involving the husbands in the process and having the Pap smear taken by female staff.

#### Community and incentive-based strategies for increasing screening uptake

*Many women don’t want to have a Pap smear. So we hold health fairs or screening campaigns where all the staff contribute money to buy food*,* noodles*,* detergent*,* and rice*,* and put together baskets. We give a basket to any woman who has a Pap smear. Every year we hold three large fairs for vaccinations*,* Pap smears*,* and other services*,* trying to convince as many people as possible.* (HCP14)

To try to convince women to participate in screening, the HCPs described outreach strategies such as health fairs and screening campaigns within primary care, particularly around Mother’s Day and Women’s Day, aimed at convincing women to get Pap smears. During the health fairs, the HCPs set up tents outside or near the facilities and gave brief educational talks to people passing by. In the interviews, they reflected on these activities and the usefulness of the health education materials traditionally employed:

*Before*,* we used to bring brochures*,* graphics*,* all those things. But our people don’t read. If even educated people don’t read*,* do you think people from rural areas will? So now what we do is offer them [women] a small gift.* (HCP16)

The quotation described HCPs hesitancy regarding the effectiveness of printed materials, but also a perceived need to use material incentives to encourage women’s participation in contexts where reading practices were perceived to be limited.

In this regard, primary care HCPs described the use of material incentives such as food baskets, also reporting that these incentives were financed out of their own pockets: *“I buy a sack of rice and sugar myself. It comes from my own pocket*,* as a doctor. With that*,* we try to convince the women […] This is a very poor community.”* (HCP9).

These food baskets often contained essential household items like rice, sugar, pasta, oil, and detergent, which made them directly useful for daily subsistence. The economic precarity of some women made these material incentives particularly persuasive: *“Unfortunately*,* the people here*,* especially in the municipality and the entire province*,* are low-income. So I think that’s why they [women] are attracted to the food baskets.”* (HCP15).

Some of the HCPs expressed generally positive views about these short-term incentive-based strategies, highlighting their perceived effectiveness in increasing screening coverage:

*We prepare food baskets and give tokens to the women who get the Pap*,* and we hold a raffle. For example*,* if there are 10 women*,* at least 5 of them get food baskets. Seeing that*,* the women agree to get the Pap*,* and we increase the coverage.* (HCP14)

However, they also expressed concerns about the conditional nature of these incentives, and reflected that women may begin to expect a reward each time, making it harder for them to participate in the long term:

*These strategies have worked*,* but the downside is that now every time we invite mothers to get a Pap smear*,* they expect something in return.* (HCP10)

These reflections highlight how providers described trying to increase screening coverage while also expressing concerns about women’s motivation and the continued use of incentives. At the same time, the HCPs acknowledged that many families lived in precarious socioeconomic conditions, and that this reliance on incentives also reflected deeper structural problems that might limit women’s ability to prioritize their health.

The HCPs also highlighted the role of community leaders and social organizations in promoting women’s participation in CC screening. They described collaboration with women’s organizations, such as the Bartolina Sisa Confederation (Bartolinas), which was considered an important strategy for sensitizing communities:

*Here in the rural area […] we work with social organizations like the Bartolinas[…] the leaders asked us for a talk on cervical cancer prevention to sensitize them[…] there have been cases that were diagnosed late[…] they help us raise awareness*,* and more women are coming to the service and getting Pap smears.* (HCP20)

The participation of male community leaders was also identified by the HCPs as key in raising awareness among men:

*We received an invitation from a man*,* who was the leader of the peasant organization*,* to give a talk on cervical cancer and speak to the husbands. He was aware that many men do not want women to go to the health centre and get a Pap smear or use contraception.* (HCP21)

Overall, the HCPs felt that the incentive-based strategies might increase women’s participation in screening, but also noted concerns about sustainability and dependence over time. They highlighted the potential of collaboration with community and social organizations, including male and female leaders.

### Distance, economics, and language shaping access

The HCPs spoke about how geographic distance, economic constraints, and language barriers intersected to limit women’s access to CC secondary prevention in Cochabamba. They framed distance as a multidimensional barrier that complicated women’s access to timely CC secondary prevention. Different aspects of this barrier were described, including the physical distance between the women’s homes and primary care facilities, the challenges of transporting samples to laboratories, and the referral pathways between primary, secondary, and tertiary care that often required multiple and time-consuming journeys in between.

Language barriers, particularly in Quechua-speaking communities, reduced understanding and engagement with both screening and referral services. The problem was compounded when HCPs working in these areas did not speak Quechua, limiting effective communication and the ability to build trust. However, when providers were able to communicate in the local language, this facilitated understanding and strengthened the patient-provider relationship.

#### Too far, too costly

*The geographic area is very spread out and rugged. Women walk two hours minimum*,* on foot. Imagine*,* two hours to get there [to the facility]*,* two hours to return. Who’s going to walk four hours just for a Pap smear?* (HCP16)

*Some women live in very remote communities where the transportation that can bring them here only operates on Mondays…”* (HCP20).

The HCPs noted that women’s ability to reach primary care services where they could have Pap smears was constrained by geographical distances and limited transportation options. In rural areas, communities were widely dispersed and located in rugged terrain, requiring long walks with infrequent transportation. An alternative to make it easier for women to access Pap smears would be to make the screening closer to where the women lived, instead of the other way round. The SAFCI model could be a way to offer such possibilities, since it was designed to bring healthcare services closer to remote communities through home visits. However, the HCPs pointed out some logistical barriers: *“Usually*,* we’ve never performed Pap smears in the community. First*,* because to reach [the community]*,* we need sterile equipment and proper facilities.”* (HCP16). At the same time, some HCPs recounted efforts to overcome these barriers, carrying examination tables into rural areas: *“We tried to organize [screening] campaigns. The [SAFCI] doctors themselves take the gynaecological examination table to the communities. We gather the patients*,* and samples are taken.” (HCP18).*

Primary care HCPs also said that due to long distances, they would accumulate screening samples for a month before transporting them to and from the cytology laboratory located in Cercado-Cochabamba municipality: *“We accumulate Paps and have to take them to the lab[…] It’s complicated for us to deliver and pick them up*,* since it’s a long way […] Sometimes road blockades also affect us”* (HCP9). Others noted that the responsibility for delivering and picking up the samples and results was not clearly assigned. In some cases, SAFCI doctors, gynaecologists, or even facility directors were the ones who ended up carrying out this task, highlighting the lack of formally established roles for this task. One provider said: *“We used to raffle among ourselves who would go and collect the results*,* but now Dr X goes [delivers the samples] and brings them back*,* but sometimes the samples get lost*,* or the results are not ready and the doctor has to go back.”* (HCP28). These practices suggest that there is an informal system for delivery and collection of samples.

This barrier was also evident in a second-level hospital in the urban area. However, in this facility the responsibility of delivering samples to the laboratory was shifted to the women themselves, adding yet another layer of burden to them:

*Now we take the Pap*,* we give it to the patients*,* and they carry it to the laboratory in Viedma [third-level hospital] […] when they deliver it [Pap sample]*,* they get a tentative date [to pick up the results]*,* three weeks*,* fifteen days[…] and if it takes longer*,* they already know it’s not our fault.* (HCP27)

Successful secondary prevention requires not only screening but also timely treatment. When women required referral from primary to secondary care level to follow up positive screening tests or precancerous lesions detected through Pap, distance became an even greater obstacle. At secondary care hospitals, the women often faced bureaucratic and time-related barriers to get an appointment with the gynaecologist, which usually forced them to make multiple trips. One provider working at a secondary-level hospital noted: *“They [women] come here to get tickets [for the waiting line] so they can schedule [an appointment] […] they can only get tickets on Fridays so […] if the patient doesn’t manage to get a ticket*,* it’s a wasted week.”* (HCP26). For women in the Andean region, where there were no secondary-level hospitals, referrals went directly to tertiary care in Cochabamba, five to six hours away. One provider described the overwhelming consequences: *“Patients don’t want to go to the city*,* they are unfamiliar with it*,* and they lack resources […] sometimes they have nowhere to sleep and end up wandering in the hospital or on the streets with their families. So they prefer not to go.”* (HCP12).

The HCPs also noted that economic and bureaucratic burdens also discouraged women from continuing treatment:

*The patients [women] tell us*,* “I don’t have money to go to Cochabamba.” […] Going to the tertiary level requires money for transportation*,* accommodation*,* and you don’t know how long they will make them [women] wait […] For example*,* a biopsy at the public gastroenterology service takes about a month*,* while in the private sector you get it in less than a week.”* (HCP2).

Hence, the HCPs perceived that geographic distance intersected with economic hardship and the limitations of the health systems, creating compound barriers for CC secondary prevention.

#### From misunderstanding to trust: the role of the Quechua language

Language also intersected with geography to create barriers, particularly in rural communities where Quechua was often the primary language spoken, especially among older women. The HCPs perceived that a lack of fluency in Quechua among healthcare staff limited women’s understanding of information related to CC preventive healthcare:

*Maybe one of the biggest problems has been language. Because most of the [healthcare] colleagues who were here didn’t speak Quechua.* (HCP22)

*If we speak to them in Spanish*,* they won’t understand*,* especially the older women. They say: “What are you talking about?”.* (HCP18)

Moreover, the HCPs stressed that even when staff attempted to use Quechua, technical or anatomical vocabulary could be inadequate if not translated into culturally appropriate and accessible terms:

*The hardest part is not knowing how to say the anatomical parts of a woman’s body in Quechua. Quechua is mostly spoken*,* not written. We try to explain in Quechua and a bit of Spanish. But how do you say “vagina” in Quechua? It would be helpful if native Quechua-speaking women could help us learn the right words*,* so we can speak accurately and they can understand.* (HCP21)

Providers also linked language barriers with reduced engagement in referral and treatment services, particularly when women had to travel to the city for follow-up care. One of them reported how navigating urban and institutional environments was especially challenging for women with limited Spanish:

*Especially older women over 40 who speak Quechua and hardly speak or understand Spanish. This is what makes it most difficult for them when they have to go to the city for follow-up appointments for treatment*,* which is why they sometimes don’t go.* (HCP26)

The HCPs noted that when they could not communicate in Quechua, this limited their patients’ understanding of procedures and engagement with care. Conversely, when they were able to speak Quechua, they perceived that this strengthened trust and facilitated follow-up: *“At first*,* I didn’t speak much Quechua*,* but after being here so long*,* now I can. I understand them*,* and they understand me.”* (HCP9). Others emphasized the practical benefits of using the local language: *“The trick is to communicate in their language. If you speak Quechua well*,* the patient understands you and comes back for follow-up.”* (HCP16). Another provider reflected on the provider-women relationship impact: *“We do better because speaking Quechua builds more trust with the patient*,* and that helps us a lot.”* (HCP10).

Overall, these perceptions of the HCPs illustrate that language proficiency was not merely a communication tool but also a potential facilitator to enhance access to CC secondary prevention.

### Caught in bureaucracy: the costs of long waits

From the HCPs’ perspective, access to CC preventive healthcare in Cochabamba was also constrained by the health system’s systemic and bureaucratic barriers. The enrolment requirements and administrative procedures of SUS often limited the reach of the programme, which created gaps in CC preventive healthcare.

#### Universal healthcare in name, bureaucratic and opportunistic in practice

*I think when they [the policymakers] designed the SUS*,* they didn’t take into consideration that Bolivians are migrants […] It’s difficult when the women require Pap and healthcare and sometimes can’t access it because they aren’t registered with the SUS in that area*,* and they have to find new insurance or pay out of pocket or go to private services.* (HCP25)

Ironically, even public-sector health workers were excluded from receiving SUS services in their own workplaces, as their insurance was tied to other providers:

*[Public] Healthcare workers who are working here in the community can’t receive care from the SUS. If they want to have a Pap smear*,* they have to go where they are insured […] So the SUS isn’t a single health system; the health system is completely divided.* (HCP30)

Administrative demands, such as the requirement to make photocopies of the women’s ID documents and proof of SUS registration, further compounded access problems. Patients who forgot or lacked these documents faced delays or denial of care. This bureaucratic rigidity represented a critical gatekeeping barrier:

*Sometimes women don’t bring their SUS card or ID card […] Sometimes they lose it*,* and if they don’t have any of these documents*,* they can’t access healthcare. Now the authorities informed us that we also need to ask them to attach a photocopy of the ID card. That complicates things*,* especially here in rural areas where we don’t have a photocopier. Some people prefer not to seek healthcare since they don’t have these documents.* (HCP27)

The HCPs highlighted that these bureaucratic demands discouraged women from seeking CC preventive healthcare. They also described considerable variability in how CC screening was implemented across health facilities. Rather than following a standardized protocol, decisions about which tests to use — Pap, VIA, HPV testing, or a combination — often depended on resource availability, local logistics, and the individual HCP’s decision:

*We perform both*,* the Pap and the HPV test. We don’t do just one. We always do both for every woman we reach. HPV was implemented here just last year… but we hear not all the facilities have it.* (HCP14)

However, others noted that VIA had been discontinued due to time constraints or insufficient training: *“We used to do VIA*,* but not anymore. It takes more time to do VIA than the Pap smear.”* (HCP11). Additionally, while policy indicated a three-year interval after two consecutive negative Pap results, some HCPs advised annual or even biannual testing: *“We tell them to do it annually*,* better if twice a year.”* (HCP16). These practices could suggest that there is an inconsistency between guidelines and implementation, meaning that screening could be non-organized and opportunistic.

Other HCPs described tensions around the exclusion of older women from the screening target group, emphasizing that some of these women had never been screened: *“In the training they told us not to take Pap smears from women over 60 or 70*,* but those patients can still have lesions[…] we have cases of elderly women with in situ cancer who’ve never had a Pap.”* (HCP20). This could suggest the need to incorporate procedures to be followed with this group of women to ensure their inclusion in screening.

#### The consequences and explanations of long waits

*When a patient goes for a Pap smear and the result is positive. I don’t know who monitors it or how. When was she referred*,* when was she treated*,* and when was the first procedure performed? I haven’t seen any regulations that mention we have to comply with timelines. I think that since there aren’t any*,* we are where we are […] The problem is the system.* (HCP21)

The HCPs highlighted that there were no established timelines or monitoring mechanisms for follow-up after abnormal Pap results, creating delays in referral, treatment, and intervention. Their statements suggested that these gaps were structural rather than individual, and they consistently identified long waiting times for Pap smear results as a critical barrier. According to the HCPs, delays often ranged from one to three months or more, undermining the effectiveness of CC prevention programs and discouraging future participation. One provider explained: *“Once we collect the sample*,* the results take a long time to arrive*,* sometimes a month*,* two months*,* or even three*,* which affects the patient’s trust. They often return and ask*,* ‘Are my results ready?’ and we have to keep telling them no.”* (HCP8). Another HCP emphasized the clinical consequences of such delays:

*The delay is one of the biggest barriers to timely treatment. Because if the result arrives three months later and we’ve detected something*,* what can we do? In these cases*,* time is gold. It’s a countdown—the later it is*,* the worse the treatment outcomes.* (HCP17)

The burden of delay also felt on health providers, who often had to deliver the results in person; this additional task was made more difficult by staffing shortages and restrictive working hours. One provider noted: *“We work until 4 in the afternoon*,* and the laboratory closes around 4:30*,* so it’s difficult to make it there in time. Also*,* there’s often no one available to go and pick up the results.”* (HCP12).

The HCPs also highlighted barriers related to having only one cytology laboratory in the Department of Cochabamba. They described the importance of the inter-municipal agreements, but noted that payments formed a barrier to timely access to cytology services. Although the laboratory at the Hospital Materno Infantil German Urquidi processed Pap smear tests free of charge under existing agreements, the Hospital del Norte required inter-municipal payments:

*We’ve been offered the option of having the tests read at the Hospital del Norte*,* but inter-municipal fees have likely prevented us from sending them there. Our municipality has an agreement with the Maternity Hospital*,* and the administrative staff say we don’t pay anything for Pap smears there.* (HCP20)

These aspects reflect how long waits for Pap results were not solely based on technical capacity, but rather on political, financial, and administrative agreements between municipalities and public institutions.

Additional structural health system reasons for the waiting included insufficient cytology staff, limited laboratory infrastructure, and centralized services. One provider said: *“There aren’t many cytologists or pathologists. Ideally there would be someone here in Mizque or in the Cono Sur region*,* but unfortunately we don’t have the professionals or the budget.”* (HCP20).

Despite efforts to advocate for improvements in the long waiting, providers noted that their concerns were not always addressed by health authorities. *“We’ve expressed our concerns to the SEDES [Departmental Health Service] many times because our Pap coverage rates don’t meet expectations. They think we’re not doing Pap smears*,* but we are. Still*,* no solutions have been provided.”* (HCP20).

The consequences of these delays were multifaceted. For example, long waiting times could result in loss to follow-up: *“We have to wait one to one and a half months for a result. Sometimes for two months. In that time*,* the patient may leave or be lost to follow-up.”* (HCP16). In cases of severe pathology, delays might directly compromise survival: *“If the result arrives two months later and it’s cancer that requires chemotherapy*,* how can we respond in time? If it’s already stage 3 or 4*,* it’s too late. We get the results too late*,* far too late.”* (HCP18).

To manage these delays, some HCPs reported both formal and informal strategies of involving NGOs and private services to speed up the return of results. One provider described a formal collaboration:

*In our municipality*,* we have a private hospital. We want to organize a Pap campaign with them […] they’ll bring all their equipment […] and they’ll give us [Pap] results within two hours […] many women could access [screening] faster.* (HCP10)

Another HCP described more informal arrangements in which they personally transported samples to private laboratories, which often involved small fees for the women:

*We inform them [women]that results take around two months […] some of them have asked us to help have the Pap read in a private service. One of the doctors takes about 5 to 10 samples per week to the private lab*,* and the results come back the following week or in two weeks. The cost is 35 bolivianos; 30 goes to the lab and the rest goes for transportation or other extra costs. Some patients can pay this amount*,* but others can’t.* (HCP2)

These strategies were perceived as effective in reducing waiting times, but they also introduced out-of-pocket costs for women, making faster access to results dependent on women’s ability to pay and potentially reinforcing existing inequities. Interestingly, the HCPs also mentioned some periods when the delivery was faster and the acceptance and demand for Paps increased: *“There was a time when results arrived faster*,* and more women came for screening. But when delays increased again*,* they stopped coming.”* (HCP11).

The HCPs also described a need for digital communication to deliver the results. One provider highlighted their experience: *“We used to coordinate with the telehealth program*,* and they sent the reports via WhatsApp. That way*,* we already knew the result and could print it here. It was advantageous.”* (HCP15). Another provider proposed a more formal implementation for a results delivery system: *“It would be good to go back to receiving results by WhatsApp. Someone there could send them digitally while the physical report arrives later. That would be a good strategy.”* (HCP12).

Overall, the HCPs’ perceptions suggested that delays not only seemed to slow down the completion of CC screening, but could also disrupt the screening loop and compromise early detection and timely care. They might also lead to the creation of informal workarounds that placed additional responsibility on both providers and women.

## Discussion

Bolivia has one of the highest CC incidence and mortality rates in Latin America, making effective secondary prevention a public health priority [1]. This study explored HCPs’ perceptions of barriers and facilitators for CC prevention in Cochabamba. The findings reflect what HCP perceive as barriers and facilitators, which is critical since their perceptions shape how services are delivered and how they interact with women.

To discuss these perceptions, we use a socio-ecological framework that provides a layered lens through which to analyse barriers and facilitators at the individual, interpersonal–community, and organizational–structural levels [20]. At the individual level, the barriers perceived by the HCPs were the women’s misconceptions about screening, fears, limited knowledge and awareness, and lack of understanding regarding the seriousness of CC. These barriers were also found in a study from Ecuador, where the participating HPCs described the women as “careless” [12]. While this may reflect widespread misinformation about CC secondary prevention, it may also be related to previous negative experiences with healthcare encounters, as described in other studies from Bolivia [18]. Most importantly, these perspectives could reflect prejudice and stereotypical thinking among HCPs, potentially contributing to how they treat women who seek CC preventive care; for example, placing on them the responsibility to access services, and blaming them if they do not use the services.

At the interpersonal-community level [20], our findings show that the HCPs perceived that barriers for women also depend on misconceptions transferred via the family, use of traditional medicine, and gender norms that reinforce the male partner’s control. Male partners’ influence on screening uptake has also been identified in other settings, such as Indonesia and Uganda [28, 29]. The HCPs in our study perceived that women sometimes even feared intimate partner violence if they attended screening. Intimate partner violence is a widespread problem for women in Bolivia [30].

The strength of the SAFCI model is that it involves healthcare professionals coming to the communities and performing close follow-up with people living in these rural and more remote areas. In countries such as Canada, Australia, and Peru, similar models have managed to increase access to CC screening through mobile clinics and platforms, such as ships that transport equipment and HCPs to perform HPV screening services in hard-to-reach communities and among Indigenous peoples, highlighting the importance of incorporating geospatial and transportation factors into screening program designs [31–33). This approach is not fully integrated within the Bolivian SAFCI model, which may be a missed opportunity to increase screening coverage, especially for women who live far away from healthcare facilities and in Indigenous communities.

A perceived facilitator was the use of the Quechua language during healthcare encounters. Primary care providers perceived that women with Quechua as their first language had more confidence and better understood the diagnosis when they explained it in Quechua, thus recognizing the importance of learning to speak this language. Speaking an Indigenous language is mandatory for public workers in Bolivia [34], but this mandate is not fully implemented. Another study found that lack of access to linguistically appropriate healthcare was a prevalent theme in the provision of healthcare services [35]. The use of Indigenous languages for the development and dissemination of health education messages has been recognized and implemented in other countries, such as Australia and Peru [36, 37], but remains limited in the Bolivian context. More research is needed to explore the implementation of Indigenous languages in the provision of CC secondary prevention.

The most commonly mentioned facilitator at this level was the use of material incentives, such as offering food baskets to persuade women to be screened during health campaigns and fairs. However, the providers also expressed a critical view of these practices. These initiatives seemed to prioritize increasing the number of women screened rather than ensuring timely diagnosis, treatment, or follow-up, which is where women face the biggest challenges related to access [13, 38, 39].

At the organizational-structural level [20], HCPs reported inviting women to undergo screening during routine healthcare visits for other purposes, such as contraceptive care or prenatal check-ups, in some cases offering Pap smears as frequently as twice a year. This practice is not in accordance with national guidelines, and suggests a non-organized and opportunistic screening; this has also been found in other settings, such as Ecuador [12]. The findings indicate a lack of population-based cervical screening, which has been widely implemented in other settings including Sweden and recently in Brazil [40, 41]. Organized CC screening can be used to invite and remind targeted women to be screened at regular intervals, and identify those not accessing screening. Bolivia has recently implemented the Nominal Vaccination Registry (RNV in Spanish), an individual registry that allows monitoring of individual vaccination programs [42]. A similar registry could be a potential facilitator to improve CC screening, and follow-up.

At the organizational-structural level [20], the HCPs described a lack of clear roles and responsibilities for sending tests and getting back the Pap results. Instead, informal systems were used. In addition, cytology reading was centralized in a single laboratory responsible for almost the entire department, resulting in significantly delayed results, mistrust of CC screening, and reluctance to engage screening due to long waiting times, an issue also reported in Peru [13]. Although a few municipalities have developed their own laboratories, political, financial, and administrative barriers, particularly inter-municipal payment requirements, hinder timely Pap results. Future research could focus on analysing the policies, infrastructure, and workforce of CC prevention to inform capacity-building efforts, as previously examined in other settings such as Belize [43]. The application of the ecological model allowed us to see that HCPs have a complex understanding of women’s access to secondary prevention, identifying barriers at all levels. However, it also makes it prevalent that the facilitators and solutions they described are primarily located on the individual level (focusing on women) and in the screening part of secondary prevention. Conversely, solutions focusing on the healthcare system and on the steps that follow after screening were presented as crucial but also harder to implement, beyond isolated initiatives.

While Levesque [19] conceptualizes access as a sequence of stages, from the perception of needs to the use of services and achievement of outcomes, we propose to include a cyclic aspect of access to recognize the process required when it comes to CC secondary prevention; access to screening is not sufficient, unless it is followed by timely access to results and then appropriate treatment. Access to CC secondary prevention involves repeated interactions: screening, diagnosis, treatment of precancerous lesions, and cancer care. When women do not obtain access to screening or timely results, the entire cycle can be disrupted. As screening requires repeated attendance every few years, many women may not return. Additionally, as we have shown, understanding access only through chronological stages is insufficient; it also has to take into account the circularity and the barriers at different layers (individual, interpersonal, community, organizational, and policy) as discussed above. If both the circular chronological stages and the layered barriers are considered in strategies to improve CC secondary prevention, it is not only clinical pathways that must be improved. Success also requires targeting the structural conditions that shape women’s ability to engage with care, ultimately contributing to more equitable access.

### Limitations

This study has some limitations. First, the study was conducted in Cochabamba, which may limit the transferability of findings to other regions of Bolivia with different health system organization, geographic challenges, or cultural norms. However, the large and diverse sample in terms of urban, rural, and remote areas is a strength, and can be useful for other contexts with similar demographics. Finally, while we identified structural challenges such as centralization of laboratories and fragmented referral systems, the study did not include direct observation of health services, and moreover did not compare the perspectives of healthcare professionals with those of the women who use the services.

### Conclusions and implications

This study explored HCPs’ perceptions of barriers and facilitators for CC secondary prevention in Cochabamba, Bolivia. The participating HCPs identified multiple interconnected barriers across the individual, interpersonal–community, and organizational-structural levels. At the individual level, the providers perceived information gaps among women, while at the interpersonal-community level, they perceived barriers in terms of family-transferred misconceptions, gender norms, and women’s fear of intimate partner violence if they attended screening. A perceived facilitator was the use of the Quechua language during healthcare encounters and the implementation of material incentives. At the organizational-structural level, the HCPs reported a lack of clarity in CC screening practices regarding which women should be screened and how often, noting the presence of some elderly women who had never been screened. They also described a screening system that had cytology services mainly centralized in one laboratory, and unclear responsibilities for healthcare staff in the delivery and collection of samples. In addition, they reported bureaucratic and administrative barriers that, in their view, limited access to screening and timely results, leading to long waiting times which generated mistrust and reluctance to undergo CC screening.

CC secondary prevention depends on the successful completion of multiple interconnected cycles of access, including screening, timely results, diagnosis, treatment, and repeated CC screening. Addressing only one part, such as screening, is insufficient and risks disrupting the entire preventive cycle. Strengthening CC secondary prevention to achieve equitable access therefore requires integrated strategies that support the continuity of these cycles while simultaneously addressing barriers across the individual, interpersonal, organizational, and structural levels.

## Supplementary Information

Supplementary Material 1.

Supplementary Material 2.

## Data Availability

The data that support the findings of this study are not openly available but are available from the corresponding author upon reasonable request.

## Abbreviations

CC: Cervical cancer
HCP: Healthcare provider
HPV: Human papillomavirus
SAFCI: Intercultural Community Family Health Model
SUS: Bolivian universal healthcare coverage
VIA: Visual inspection with acetic acid
