# Supplementary material for: Barriers and facilitators in access to cervical cancer secondary prevention in Cochabamba, Bolivia: a qualitative study of healthcare providers’ perceptions
Source: BMC Prim Care. 2026 Jun 15;27:233. doi: 10.1186/s12875-026-03422-2 (PMC13270897; doi:10.1186/s12875-026-03422-2)
Supplement: Supplementary file 1 — Supplementary Material 1. [file 12875_2026_3422_MOESM1_ESM.docx]

**HEALTHCARE PROVIDERS INTERVIEW GUIDE**

Good morning/good afternoon

My name is…...from………I'm conducting research to explore the challenges and opportunities in the provision of healthcare in the secondary prevention (screening and early treatment) of cervical cancer from the perspective of healthcare providers working in public health centers and hospitals in the Department of Cochabamba, Bolivia.

Your experiences as healthcare providers and points of view are extremely valuable for this study. This information will allow us to analyze and build strategies to strengthen cervical cancer prevention and, in some way, help improve access for women who are affected.

Before we begin, I would like to remind you that this interview will be recorded. You may refuse to answer any questions or stop the interview if you feel uncomfortable. You may also withdraw your participation at any time. Do you have any questions for me*?

* Take notes about the concerns raised by the participants.

| **Sociodemographic characteristics** | |
| --- | --- |
| 1. **Age** |  |
| 1. **Gender** |  |
| 1. **Level of Education** |  |
| 1. **Healthcare facility** |  |
| 1. **Healthcare Network** |  |
| 1. **Municipality** |  |
| 1. **Macro region to which the healthcare facility belongs** |  |
| 1. **Role in the healthcare facility** |  |
| 1. **Time working in this role** |  |
| 1. **Years of professional training experience** |  |

| **Research aim** | Explore healthcare providers' perceptions of barriers and facilitators for women’s access to cervical cancer secondary prevention in Cochabamba, Bolivia. |
| --- | --- |
| **Interview Components** | **Questions** |
| **1. Perception of the response of the public healthcare system in relation to cervical cancer** | 1. What role are you playing in this healthcare facility/hospital?   We will talk about the functioning of the health system in relation to the entire cervical cancer screening, from sample collection to the delivery of results   1. How do you experience cervical cancer screening activities at your workplace? 2. From your experience, what difficulties have you encountered in your activities related to cervical cancer screening (from sample collection, processing, to delivering results)? Tell us about some experiences with this.   Follow-up question: I got interested in (a particular experience that the interviewer mentioned)   1. What strategies are being used to try to overcome these barriers?   Follow-up question: Ideally, what aspects should be improved in this situation in primary care?  Then the healthcare system… |
| **2. Encounter between healthcare providers and women** | Now we will talk about your experiences in encountering and recruiting women for screening   1. What experiences can you comment on the activities that this healthcare center has undertaken to increase screening coverage?   Follow-up question: If the person has any particular experience: What is your motivation to do these activities/ apply the strategies?   1. From your perspective, what do you think could be done differently, and what strategies could be implemented in the public system to improve the screening coverage? |
| **3. Communication between healthcare providers and women** | Now we will talk about communication with women   1. How have you seen/what do you think about women's understanding of Pap smears and cervical cancer?   Follow-up question:  What strategies do you use to help women understand you better?   1. What is your opinion about the communication between healthcare providers and women when communicating Pap results, diagnosis, and treatment?   Follow-up question:  What would you suggest to improve communication with patients and their understanding of cervical cancer and PAP/VIA? |
| **4. Perception of the obstacles that women face** | Now we will talk about your experiences and how you perceive the barriers that limit women from being screened   1. From your experience, what difficulties have you encountered in getting women to accept a PAP/VIA in your healthcare facility/hospital? If the limitations of the healthcare system were resolved, what aspects would influence women to want or not to get a Pap smear?   Follow-up question:  What other barriers could be limiting women when it comes to getting the Pap smear?   1. From your point of view, which groups of women are in a situation of greater vulnerability and do not go to screening? |
| **5. Final question** | 1. Is there anything you would like to add or any important points that we have not discussed? |
| **Stop recording** | How did you experience the interview?  Guideline: Thank the interviewee for his/her time and collaboration |
