# Supplementary material for: Barriers and facilitators in access to cervical cancer secondary prevention in Cochabamba, Bolivia: a qualitative study of healthcare providers’ perceptions
Source: BMC Prim Care. 2026 Jun 15;27:233. doi: 10.1186/s12875-026-03422-2 (PMC13270897; doi:10.1186/s12875-026-03422-2)
Supplement: Supplementary file 2 — Supplementary Material 2. [file 12875_2026_3422_MOESM2_ESM.docx]

**INFORMED CONSENT FOR HEALTHCARE PROVIDERS**

**Purpose of the study**

We would like to invite you to participate in the doctoral research: "Access to cervical cancer preventive health care in Cochabamba, Bolivia". This study is conducted by……working at………

The purpose of the study is to explore the challenges and opportunities in the provision of healthcare in the secondary prevention (screening and early treatment) of cervical cancer from the perspective of healthcare providers working in public health centers and hospitals in the Department of Cochabamba, Bolivia.

This study is not linked to the Departmental Healthcare Service. Therefore, you can participate by providing your point of view on what works well and what needs to be improved in the public healthcare facilities.

**How does this study work?**

If you would like to participate in this study, we will coordinate with you on the time and place to interview you. The interview will last between 30 minutes and 1 hour and can be conducted in a closed environment at the healthcare facility, hospital or the Faculty of Medicine of San Simon University to ensure confidentiality. The interview will be like a normal conversation between two people. The questions during the interview are open-ended and you will be asked about your point of view regarding the limitations of the public healthcare system in the detection, diagnosis, and early treatment of cervical cancer. You will be asked about your perspective on opportunities to improve and strengthen secondary prevention of this disease. You decide how much you want to say and whether you want to answer some questions.

**What will happen to your data and the information you provide?**

To participate in the study, we do not require you to give us confidential information such as your ID number, address, etc. Your identity will be kept anonymous, your name and contact details will not be mentioned in the project documents. We would like to keep your name and phone number in case we need to ask follow-up questions after the interview. We will store this information separately from the interview. The interview will be recorded and then transcribed in written form. All information provided will be encrypted and will be stored in the Faculty of Medicine's Database Center of San Simon University. Only the research team will have access to it. To present the study's results to the scientific community, we will ensure that no identifiable information will be shown.

**What are the possible risks and benefits of participating in the study?**

Your participation in this study is completely voluntary. By participating in the study, you will help us understand the challenges and opportunities in providing cervical cancer secondary prevention healthcare. Participating in the study will allow you as a participant to share your experiences, ideas, and suggestions for improving healthcare.

**How can you obtain information about the study results?**

You will be able to obtain the transcript of your interview and the study's complete results by contacting me. You will find my contact information below.

**Compensation**

You will not be compensated for participation in the study.

**Responsible**

Carla Huanca Challgua email: [carla.huanca@umu.se](mailto:carla.huanca@umu.se) c.huanca@umss.edu

**Consent to participate in the study**

I have received verbal and written information about the study, I have had the opportunity to ask questions, and I declare that I understand what it is about.

- I voluntarily agree to participate in the study "Access to cervical cancer preventive medical care in Cochabamba, Bolivia". I understand that I can decide not to participate and that I can withdraw from the study at any time.
- I agree that my personal data will be processed as described above and I understand that my confidentiality will be protected.

A signed copy of this document will be kept as proof of this.

**Place and date** **Signature**
